# Suicide prevention: Putting the person at the center

**DOI:** 10.1371/journal.pmed.1002938

**Published:** 2019-09-30

**Authors:** Vikram Patel, Pattie Pramila Gonsalves

**Affiliations:** 1 Department of Global Health and Social Medicine, Harvard Medical School, Boston, Massachusetts, United States of America; 2 Sangath, New Delhi, India

## Abstract

In September’s Editorial, Vikram Patel and Pattie Gonsalves discuss suicide prevention, the focus of World Mental Health Day, 2019.

The focus of this year’s World Mental Health Day on suicide prevention is very timely because although much is known about the epidemiology of suicide, its causes, and approaches to prevention, action and interventions are lacking across local, regional, and national levels for different population groups. In this Editorial, we discuss the key findings and recommendations arising from the relevant evidence but argue that the focus on preventing suicide must extend beyond prevention of suicide mortality to addressing the loss of hope that underlies each attempt to end a person’s life.

## Diverse patterns of suicide epidemiology

Every year, at least 800,000 people die from suicide, a figure that is likely to be a gross underestimate of the actual numbers for a variety of reasons, not least shame and discrimination. Reassuringly, the Global Burden of Disease study has indicated that the global age-standardized mortality rate for suicide decreased by a third between 1990 and 2016. However, there are large variations in the burden across countries and regions and between the sexes. Thus, the highest regional age-standardized mortality rates in 2016 were estimated for eastern Europe, followed by high-income Asia Pacific and southern sub-Saharan Africa. Rates for men were higher than for women across regions, countries, and age groups, except for the 15–19 age group, and the reduction in mortality rates over those two and a half decades was greater in women than in men [1]. Suicide can occur throughout life but is more common in young people, among whom it is the second leading cause of death globally, and in older people.

It is argued, rightly, that each act of suicide is the result of a unique interaction of sociodemographic, economic, cultural, and health-related factors, all of which occur within a broader ecological context with a range of structural factors that play out, disproportionately, across subgroups in the population. It appears that mental health problems such as depression are less commonly reported risk factors in low-income countries (as compared with high-income countries) [2] and also in young people (as compared with older people). Rapid social change, fueled in recent times by the digital world, is a potential driver for despair stemming from aspirational failure in young people. India, which is now the epicenter of suicide mortality, accounting for a third of all female and a quarter of all male suicide deaths globally, and where suicide is the single leading cause of death in young people, is an exemplar of how diverse ecological factors contribute to suicide [3]. Gender- and sexuality-based discrimination, socially conservative attitudes that prohibit romantic relationships and marriage across castes and religion, pressure to perform well academically, caste-based harassment, and sexual abuse are among the diverse triggers for suicide in India [4–6].

## The global response to suicide prevention

About 40 countries have adopted standalone suicide-prevention strategies and, although few are in low- and middle-income countries, where 79% of suicides occur, there are examples of countries like Sri Lanka that have dramatically reduced suicide mortality consequent to such plans [7]. The inclusion of suicide among the indicators of the UN Sustainable Development Goals (SDGs) should motivate all countries to implement evidence-based interventions for its prevention. The Disease Control Priorities program and WHO have documented a number of cost-effective interventions, ranging from restricting means (perhaps the most dramatic recent examples of success being pesticide control in Asia but also, conversely, the rising rates of suicide by firearm in countries like the United States that have failed to regulate access to guns) to improving access to quality mental health care, in particular for depression, alcohol abuse, and psychoses [8,9]. It is important to note, however, that such evidence-based syntheses bias against interventions that address the structural determinants that cannot be evaluated through randomized controlled trials or whose impacts on suicide have simply not been reported, such as interventions promoting gender empowerment or challenging discrimination against marginalized groups.

Thus, a “one size fits all” approach will not apply to suicide prevention. Intersectoral, coordinated, and collaborative actions that target the range of risk factors is probably the most successful public health strategy. Most of the existing national plans share a number of common elements, including “public education, responsible media reporting, school-based programs, detection and treatment of depression and other mental disorders, attention to those with drug and alcohol problems, attention to those with somatic illnesses, improved access to mental health services, improvement in assessment of attempted suicide, crisis intervention, work and unemployment policy, training of health professionals, and reduced access to lethal means” [10]. Meaningful reductions in suicides will depend on the successful implementation of these multicomponent recommendations. Alongside health and mental health professionals and organizations, there are critical roles for families, schools, businesses, community and religious leaders, the social welfare and judicial systems, and the media. National and regional governments need to mobilize and coordinate action in these sectors and define a contextually appropriate response through a national strategy that aims for at least a 10% reduction in the suicide rate initially, as agreed in the WHO Mental Health Action Plan 2013–2020, and a reduction by one-third by 2030, as proposed in the SDGs.

## Beyond deaths: The person who has lost hope

Although the decrease in suicide mortality has been substantial, if current trends continue, only 3% of 188 countries will attain the SDG target to reduce suicide mortality by one-third between 2015 and 2030 [1]. Further, it is important to note that our attention must also address suicide attempt rates, which have, if anything, increased whereas suicide mortality has fallen. A recent systematic review, for example, that reviewed self-harm in 597,548 participants from 41 countries reported an overall lifetime prevalence of 16.9% (95% CI 15.1–18.9), with rates increasing to 2015. Self-harm was strongly associated with suicide attempts, suggesting that these are highly correlated behaviors. In contrast to mortality, girls were more likely to self-harm (risk ratio 1.72, 95% CI 1.57–1.88) [11]. In another recent study with over 6,500 adults randomly sampled from districts in four low- and middle-income countries, the proportion of people who reported suicidal ideas or plans was up to 10 times greater than the proportion of attempts [12]. Thus, although suicide mortality is falling, self-harm behavior may be on the rise, suggesting that efforts to reduce the lethality of the attempt are succeeding, but there seems to be little respite from the proximal determinant of those attempts, the suicidal behavior itself.

This discrepancy highlights that, while we focus on systems and sectors, we must not lose sight of the individual. A person-centered approach, one of the guiding principles of universal health coverage for mental health problems [13], is critically relevant to suicide prevention. Regardless of the complex interplay of factors that lead an individual to commit suicide, there are common proximal pathways that precede the final act, and these are dominated by core emotions of hopelessness, shame, and anger, compounded by an overwhelming sense of helplessness. The most frequent reason for self-harm is relief from such distressing thoughts or feelings [11]. We must address these intensely private experiences that cloud the minds of individuals who are contemplating, planning, and ultimately carrying out an action that will imperil their lives. This will require addressing these emotions head-on, for example, through public engagement campaigns that create safe and open spaces for discussing mental health and by making help-seeking for distress as “normal” as for any physical health problem (as an example, the “It’s OK to Talk” campaign targeting young people in India) [14]; creating opportunities to empower children and young people to improve the situation in schools and learn core life skills to manage disappointments and prevent impulsive actions (for example, the Learning Together universal school-based intervention for 11- to 16-year-olds, which uses a restorative approach to address youth bullying and aggression) [15]; ensuring affordable and accessible quality services for interventions that address the social determinants that are often the major risk factors of both poor mental health and suicide (as an example, “headspace” centers in Australia that provide point-of-service access to young people aged 12–25) [16]; and engaging peers in support-based programs (for example, “7Cups of Tea,” an online platform that provides free volunteer-based emotional support) [17].

The important and timely theme of this year’s World Mental Health Day reminds us that every country and every community needs to recognize and act on the rich evidence that already exists, showing that suicide is at the same time one of the most acute and one of the most tractable of all mental health–related outcomes. At the heart of this action is promoting the knowledge that all suicides are potentially preventable and, with appropriate skills, we can take the opportunity to reach out to someone we are near—a relative, a friend, a colleague—in a way that could change the trajectory of their life. Although preventing suicide is everyone’s business, it is the responsibility of the state to provide stewardship for coordinated actions across sectors and to ensure that commitments to reduce suicide, and self-harm more generally, are realized.
